# Regulation of CD4^+^ T Cells by Pleural Mesothelial Cells via Adhesion Molecule-Dependent Mechanisms in Tuberculous Pleurisy

**DOI:** 10.1371/journal.pone.0074624

**Published:** 2013-09-19

**Authors:** Ming-Li Yuan, Zhao-Hui Tong, Xiao-Guang Jin, Jian-Chu Zhang, Xiao-Juan Wang, Wan-Li Ma, Wen Yin, Qiong Zhou, Hong Ye, Huan-Zhong Shi

**Affiliations:** 1 Department of Respiratory and Critical Care Medicine, Beijing Chaoyang Hospital, Capital Medical University, Beijing, China; 2 Department of Respiratory and Critical Care Medicine, Union Hospital, Tongji Medical College, Huazhong University of Science and Technology, Wuhan, China; 3 Department of Pathophysiology, Tongji Medical College, Huazhong University of Science and Technology, Wuhan, China; 4 Center of Medical Research, Beijing Institute of Respiratory Diseases, Beijing, China; Chinese Academy of Sciences, China

## Abstract

**Background:**

Intercellular adhesion molecule-1 (ICAM-1) and vascular cell adhesion molecule-1 (VCAM-1) have been demonstrated to be expressed on pleural mesothelial cells (PMCs), and to mediate leukocyte adhesion and migration; however, little is known about whether adhesion molecule-dependent mechanisms are involved in the regulation of CD4^+^ T cells by PMCs in tuberculous pleural effusion (TPE).

**Methods:**

Expressions of ICAM-1 and VCAM-1 on PMCs, as well as expressions of CD11a and CD29, the counter-receptors for ICAM-1 and VCAM-1, respectively, expressed on CD4^+^ T cells in TPE were determined using flow cytometry. The immune regulations on adhesion, proliferation, activation, selective expansion of CD4^+^ helper T cell subgroups exerted by PMCs via adhesion molecule-dependent mechanisms were explored.

**Results:**

Percentages of ICAM-1-positive and VCAM-1‒positive PMCs in TPE were increased compared with PMC line. Interferon-γ enhanced fluorescence intensity of ICAM-1, while IL-4 promoted VCAM-1 expression on PMCs. Percentages of CD11a^high^CD4^+^ and CD29^high^CD4^+^ T cells in TPE significantly increased as compared with peripheral blood. Prestimulation of PMCs with anti‒ICAM-1 or ‒VCAM-1 mAb significantly inhibited adhesion, activation, as well as effector regulatory T cell expansion induced by PMCs.

**Conclusions:**

Our current data showed that adhesion molecule pathways on PMCs regulated adhesion and activation of CD4^+^ T cells, and selectively promoted the expansion of effector regulatory T cells.

## Introduction

Tuberculosis remains a major global health problem and is one of the leading causes of morbidity and mortality from infection. One third of the world’s population are thought to be infected with *Mycobacterium tuberculosis* (MTB), and in 2011, 8.7 million new active tuberculosis cases were reported with 1.4 million deaths from MTB infection [1]. In China, the prevalence of active, smear-positive, bacteriological positive pulmonary tuberculosis in 2010 was 459/100,000, 66/100,000, 119/100,000, respectively [2].

Tuberculous pleural effusion (TPE) results from MTB infection of the pleura and is characterized by an intense chronic accumulation of inflammatory cells at the disease site. An accumulation of lymphocytes, especially CD4^+^ T cells, in TPE has been well documented [3]{Porcel, 2009 #1}. More and more studies have reported that several Th subsets, such as Th1 cells [4], Th17 cells [5], and regulatory T cells (Tregs) [6], etc. were involved in the pathogenesis of TPE, with various Th cells maintaining delicate balance. However, mechanisms of the dynamic balance of Th cells in TPE were still unclear.

Pleural mesothelial cells (PMCs), presented in a single layer covering each pleural membrane, are exposed to a microenvironment with high levels of cytokines and chemokines during infection, initiating and propagating an inflammatory reaction by coordinating the other kinds of inflammatory cells [7]. Our recent studies have demonstrated that PMCs derived from TPE expressed high levels of HLA-DR and co-stimulatory molecules, CD80/CD86, and functioned as antigen presenting cells to promote proliferation and differentiation of naïve CD4^+^ T cell in the presence of MTB specific antigens [8,9].

Intercellular adhesion molecule-1 (ICAM-1) and vascular cell adhesion molecule-1 (VCAM-1) are known to interact with their major counter-receptors, lymphocyte function-associated antigen-1 (LFA-1, CD11a/CD18) and very late antigen-4 (VLA-4, CD49d/CD29), respectively; such interactions greatly increase the avidity of T cells and antigen presenting cells, and thus modulate the signal transduction pathways that control complex cell functions, including T cell activation and differentiation [10]. It has been reported that under stimulation of bacillus calmette-guérin or asbestos, PMCs were induced to express ICAM-1 and VCAM-1, through which PMCs facilitated monocyte transmigration or leukocyte adhesion [11,12]. In the present study, we were promoted to explore the regulations of PMCs via adhesion molecule-dependent mechanisms on adhesion, proliferation, activation, and selectively polarization of CD4^+^ T cells.

## Materials and Methods

### Subjects

The study protocol was approved by our Institutional Review Boards for human studies of Capital Medical University, Beijing, China; and Tongji Medical College, Wuhan, China; and informed written consent was obtained from all subjects. Twelve anti‒HIV Ab negative patients (sex, 8 male and 4 female; age, 41.4 ± 3.8 yr) were proven to have TPE, as evidenced by the presence of MTB in pleural fluid or by demonstration of granulomatous pleurisy on pleural biopsy specimen in the absence of any evidence of other granulomatous diseases. After anti-tuberculosis chemotherapy, the resolution of TPE and clinical symptoms was observed in all patients. At the time of sample collection, none of the patients had received any anti-tuberculosis therapy, corticosteroids, or other nonsteroid anti-inflammatory drugs.

### Sample Collection and Processing

Five hundred to 1,000 ml of TPE samples from each patient were collected in heparin-treated tubes, through a standard thoracocentesis technique within 24 h after hospitalization. Twenty milliliters of blood were drawn simultaneously. TPE specimens were immersed in ice immediately and were then centrifuged at 1,200 g for 5 min. The cell pellets of TPE were resuspended in HBSS, and mononuclear cells were isolated by Ficoll-Hypaque gradient centrifugation (Pharmacia, Uppsala, Sweden) to determine the T cell subsets within 1 h.

### Flow Cytometry

The expressions of markers on T cells from TPE and blood were determined by flow cytometry as previously described [8,9] after surface or intracellular staining with Abs conjugated with FITC, PE, PE-Cy7, PerCP, PerCP-Cy5.5, APC, or eFluor 660. These human Abs included anti–CD3, –CD8, –CD45RA, –CD11a, –CD29, –IL-22, –IL-17, –IL-9, –IFN-γ, and –FoxP3 mAbs, which were purchased from BD Biosciences (Franklin Lakes, NJ), eBioscience (San Diego, CA), or R&D systems (Minneapolis, MN). Intracellular staining for IL-17, IL-4, or IFN-γ was performed on T cells stimulated with phorbol myristate acetate (50 ng/ml; Sigma-Aldrich, St. Louis, MO) and ionomycin (1 µM; Sigma-Aldrich) in the presence of GolgiStop (BD Biosciences) for 5 h, and then stained with anti–IL-22, -IL-17, –IL-9, –IFN-γ, or –Foxp3 mAb conjugated with PE, PerCP-Cy5.5, or PE-Cy7 (BD Biosciences or eBioscience). Appropriate species matched Abs served as isotype control. To explore the expressions of adhesion molecules on PMCs, anti–ICAM-1 and –VCAM-1 mAbs (eBioscience) conjugated with APC or PE were used. Flow cytometry was performed on a FACS Canto II (BD Biosciences) and analyzed using BD FCSDiva Software and FCS Epress 4 software (De Novo Software, Los Angeles, CA).

### Cell Isolation

CD4^+^ T cells were isolated by MACS based on positive selection using the CD4^+^ T cell isolation kit II (Miltenyi Biotec, Bergisch-Gladbach, Germany) according to the manufacturer’s instructions. The purity of CD4^+^ T cells was > 97%, as measured by flow cytometry.

For isolating PMCs, the cell pellets of TPE were resuspended in RPMI-1640 (Gibco, Invitrogen, Carlsbad, CA) containing 20% heat-inactivated fetal bovine serum (FBS; Gibco), 20 ng/ml epidermal growth factor (R&D systems), which didn’t affect expressions of ICAM-1 and VCAM-1 on PMCs (Figure S1), and 50 µg/ml gentamycin. The cells were seeded into 25-cm^2^ flasks at a density of 1 × l 0^4^ cells/cm^2^ and placed in an incubator at 37 °C in 5% CO_2_. After 24 h the monolayers were washed with HBSS to remove nonadherent cells and fresh media was added. The monolayers were monitored until confluent (7‒10 d), then trypsinized, and subcultured for 5 to 6 passages. After each passage the cells grew to confluence within 4‒5 d. In general, PMCs could be maintained for 6 to 7 passages before they became senescent.

In addition, a non-malignant transformed mesothelial cell line (Met5A cell), purchased from ATCC (Manassas, VA, USA) was used as a control as previously reported [13].

### Stimulation of PMCs with Cytokines or TPE supernatants

We cultured PMCs in medium alone, or in the presence of IFN-γ (50 ng/ml; R&D Systems), IL-4 (100 ng/ml; R&D Systems), IL-17 (100 ng/ml; R&D Systems), TGF-β (5 ng/ml; R&D Systems), MTB-specific peptide of early secretory antigenic target-6kDa/culture filtrate protein-10 (ESAT-6/CFP-10) (10 µg/ml, State Key Laboratory of Agricultural Microbiology, Huazhong Agricultural University, Wuhan, China). We also cultured PMCs in TPE (50% volume) in the presence or absence of anti‒IFN-γ mAb (10 µg/ml; eBioscience) or anti‒IL-4 mAb (10 µg/ml; eBioscience). After 48 h, expressions of ICAM-1 and VCAM-1 on PMCs were determined by flow cytometry.

### CD4^+^ T Cell-PMC Adhesion Assays

PMCs were cultured in 48-well plates to form confluent monolayers and cocultured with autologous CD4^+^ T cells purified from TPE at the ratio of 1 : 10. In some cocultures, PMCs were preincubated with anti‒ICAM-1, ‒VCAM-1 mAb, or irrelevant isotype control IgG1 (10 µg/ml each) for 4 h. After 48 h of incubation at 37 °C in 5% CO_2_, non-adherent cells were removed by three gentle washes with PBS. Cells were fixed with 4% paraformaldehyde, and underwent Wright staining, then were viewed and photographed under a digital microscope (Olympus BX51; Olympus, Tokyo, Japan). The percentages of CD4^+^ T cell-PMC rosette formation were determined. Rosette formation was defined as ≥ 2.5 CD4^+^ T cells attached to each PMC. 200 PMCs were counted blindly in each experiment.

**Figure 1:**
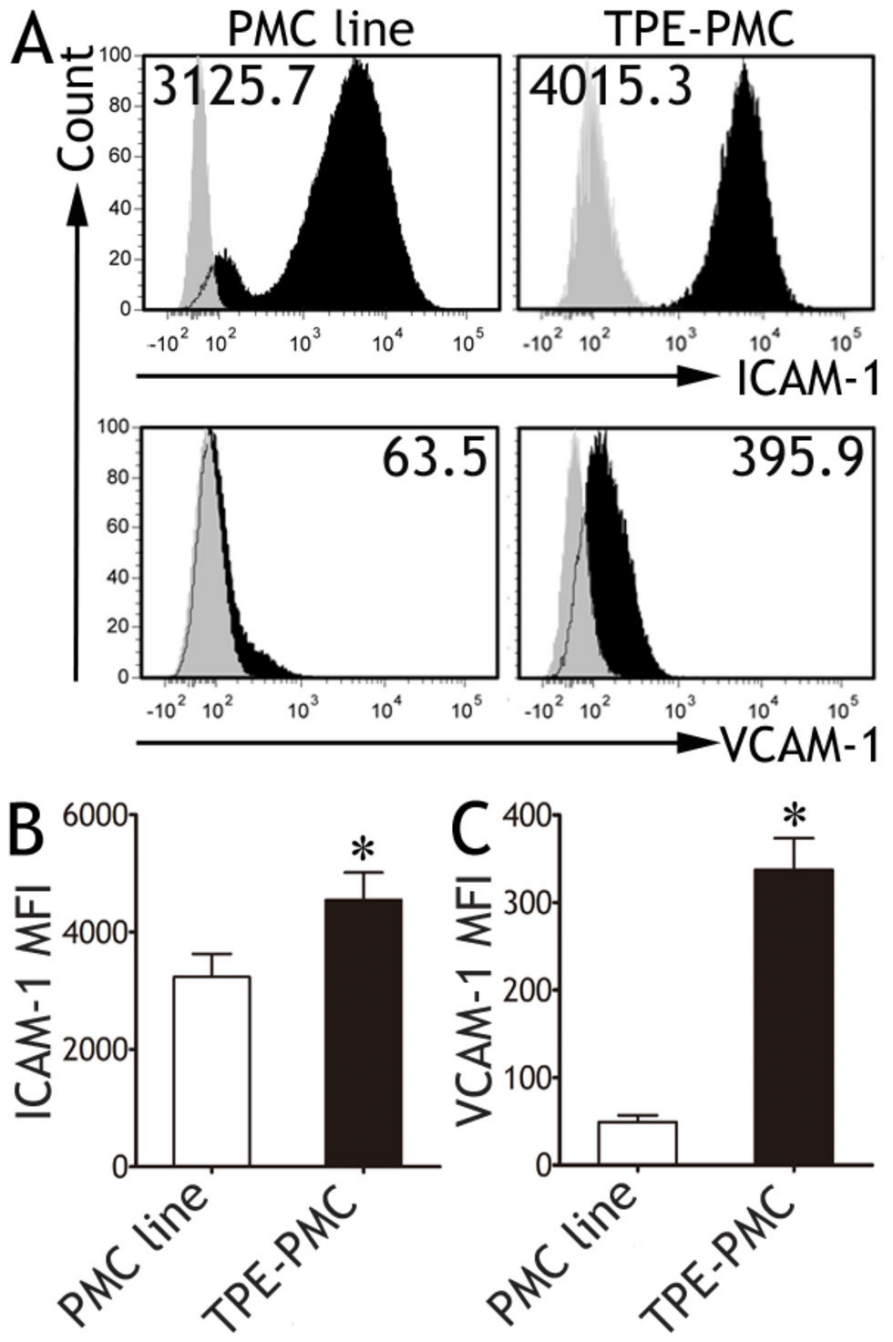
Expressions of intercellular adhesion molecule-1 (ICAM-1) and vascular cell adhesion molecule-1 (VCAM-1) on pleural mesothelial cells (PMCs). A non-malignant transformed mesothelial cell line Met5A cell (PMC line, n = 12) or PMCs derived from tuberculous pleural effusion (TPE, n = 12) were stained using anti‒ICAM-1, ‒VCAM-1 mAb, or isotype control IgG. (A) Representative flow cytometric histogram plots show ICAM-1 and VCAM-1 expressions on PMCs. Light gray histograms indicate isotype controls. (B. C) Comparisons of mean fluorescence intensity (MFI) of ICAM-1 and VCAM-1 on PMCs. The results are reported as mean ± SEM. The comparisons were determined by Mann Whitney U test. *p < 0.05 compared with PMC line.

### CD4^+^ T Cell Selective Differentiation Mediated by PMCs

PMCs were cocultured with autologous CD4^+^ T cells at a ratio of 1 : 5 in RPMI-1640 supplemented with penicillin (100 U/ml), streptomycin (100 µg/ml), L-glutamine (2 mM), HEPES (10 mM), 10% type AB human serum in the presence or absence of anti‒CD3 mAb (OKT; 1 µg/ml) in flat bottomed 48-well plates. CD4^+^ T cells (2 × 10^5^) cultured alone or in the presence of anti‒CD3 mAb (OKT; 1 µg/ml) served as controls. In some experiments, PMCs were preincubated with anti‒ICAM-1, ‒VCAM-1 mAb, or control IgG1 (10 µg/ml each) for 4 h. 5 d later, suspended T cells were collected and washed three times. For proliferation and activation, CD4^+^ T cells were intracellular stained with anti‒Ki-67 mAb or surface stained with anti‒CD25 mAb; for cytokine production, CD4^+^ T cells were restimulated with phorbol myristate acetate (50 ng/ml; Sigma-Aldrich, St. Louis, MO) and ionomycin (1 µM; Sigma-Aldrich) in the presence of GolgiStop (BD Biosciences) for 5 h. The expressions of Ki-67, CD25, IL-22, IL-17, IL-9, IFN-γ, and FoxP3 were determined with flow cytometry in gated on CD3^+^ and CD8^−^ T cells.

**Figure 2:**
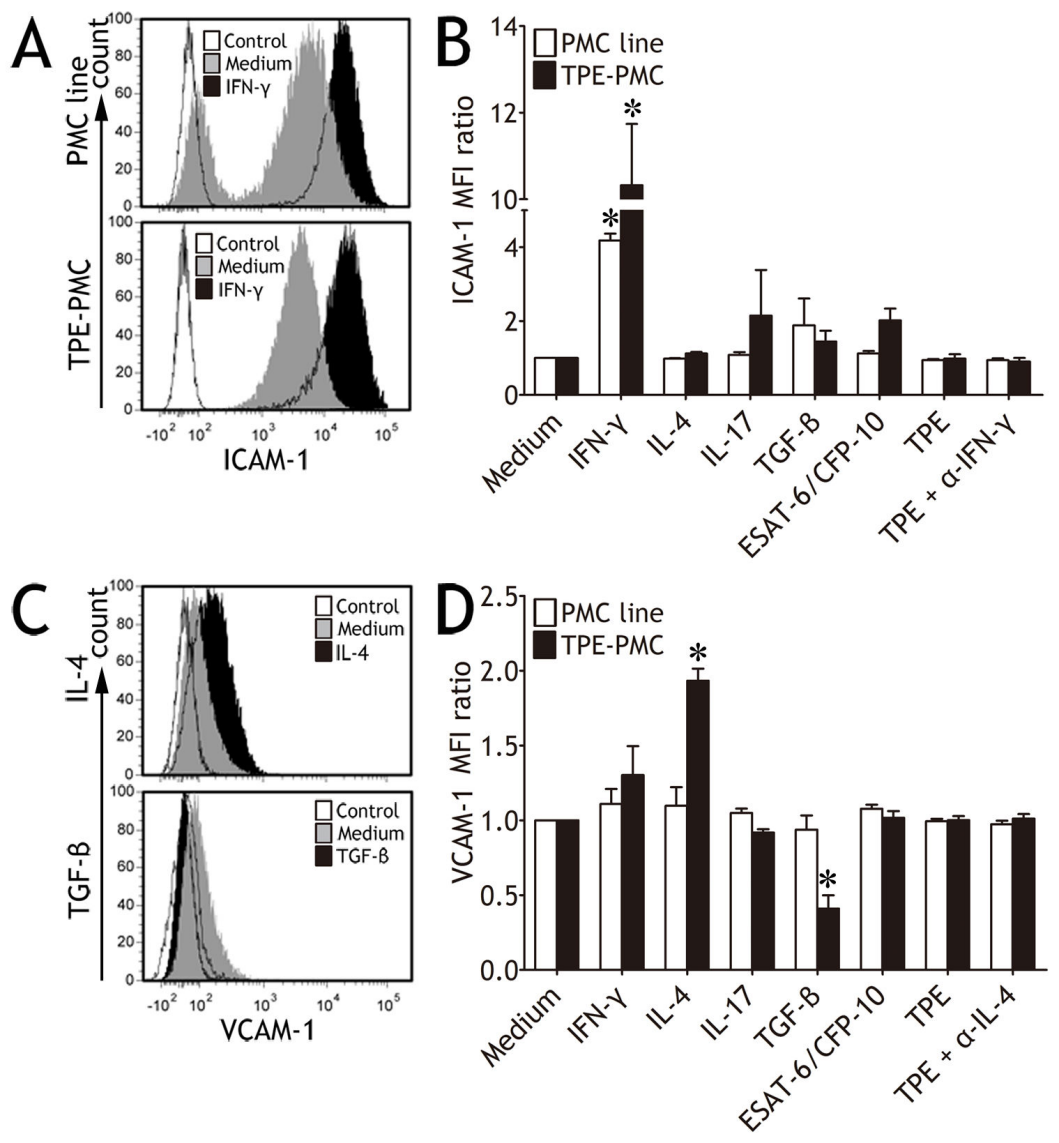
Regulation of expressions of intercellular adhesion molecule-1 (ICAM-1) and vascular cell adhesion molecule-1 (VCAM-1) on pleural mesothelial cells (PMCs) *in vitro*. A non-malignant transformed mesothelial cell line Met5A cell (PMC line) or PMCs derived from tuberculous pleural effusion (TPE) were cultured in medium alone, designated cytokines, tuberculosis-specific antigens (ESAT-6/CFP-10), or 50% volume TPE in the absence or presence of anti‒IFN-γ or ‒IL-4 mAbs. After 48 h, PMCs were stained and detected by flow cytometry. (A) Representative cytometric histograms show ICAM-1 expression on PMC line and PMCs from TPE cultured in medium alone or stimulated by IFN-γ. White histograms indicate isotype controls. (B) Comparisons of ICAM-1 MFI ratios of PMCs are shown. ICAM-1 fluorescence intensity (MFI) ratios were calculated by dividing MFI of each group by MFI of control group, respectively. (C) Representative cytometric histograms show VCAM-1 expression on PMCs from TPE cultured in medium alone, stimulated by IL-4 or TGF-β. White histograms indicate isotype controls. (D) Comparisons of VCAM-1 MFI ratios of PMCs are shown. In panels B and D, the results are reported as mean ± SEM from 5 independent experiments. The comparisons were determined by Kruskal-Wallis one-way analysis of variance on ranks. *p < 0.05 compared with the corresponding controls.

### Statistics

Data are expressed as mean ± SEM. For variables in TPE and in corresponding blood, paired data comparisons were made using a Wilcoxon signed-rank test, while unpaired data comparisons were made using a Mann Whitney U test. Comparisons of the data between different groups were performed using a Kruskal-Wallis one-way analysis of variance on ranks. Analysis was completed with SPSS version 16.0 Statistical Software (Chicago, IL), and p values of less than 0.05 were considered to indicate statistical significance.

## Results

### ICAM-1 and VCAM-1 Expressed on PMCs

Biochemical and cytological characteristics in TPE are illustrated in Table 1. Subjects with tuberculosis showed a large proportion of these cells were lymphocytes, with some neutrophils, macrophages, and mesothelial cells.

**Table 1:** Biochemical and cytological characteristics in tuberculous pleural effusion.

Variables	Results
Protein	42.2 ± 6.6 g/L
Lactate dehydrogenase	476.2 ± 50.5 IU/L
Adenosine deaminase	64.3 ± 6.4 U/L
Total cell counts	3.52 ± 1.3 × 10^9^/L
**Differential cell counts**	
Lymphocytes	74.9 ± 5.3%
Neutrophils	8.8 ± 1.3%
Macrophages	13.8 ± 1.3%
Mesothelial cells	2.5 ± 0.4%

Consistent with Nasreen and colleagues’s findings [11], we noted that almost all PMCs isolated from TPE expressed ICAM-1, and the mean fluorescence intensity (MFI) was higher than that of Met5A cells (PMC line) (4544 ± 468.9% *versus* 3235 ± 392.6%, both n = 12, p = 0.0304, Mann Whitney U test) (Figure 1A and 1B). We also noted that TPE derived-PMCs expressed moderate levels of VCAM-1(337.3 ± 36.2%), which was also significantly higher than PMC line did (49.3 ± 7.6%, p < 0.001). Additionally, PMCs derived from transudative pleural effusion expressed lower levels of ICAM-1 and VCAM-1 than TPE derived-PMCs, though it was not statistically significant (Figure S2).

### Regulation of ICAM-1 and VCAM-1 Expressions on PMCs

As shown in Figure 2A and 2B, IFN-γ was the only one cytokine that notably enhanced MFI of ICAM-1 expression on PMCs isolated from TPE as well as PMC line. It was also found that MTB-specific antigens ESAT-6/CFP-10 did not significantly increase ICAM-1 MFI, neither did TPE. On PMCs from TPE, IL-4 markedly upregulated VCAM-1 expression, while TGF-β downregulated VCAM-1 expression (Figure 2C and 2D). In addition, ESAT-6/CFP-10 did not affect VCAM-1 expression on TPE derived-PMCs (Figure 2D). In terms of VCAM-1 expression, PMC line did not react as vigorously to stimulations as PMCs from TPE did (Figure 2D).

**Figure 3:**
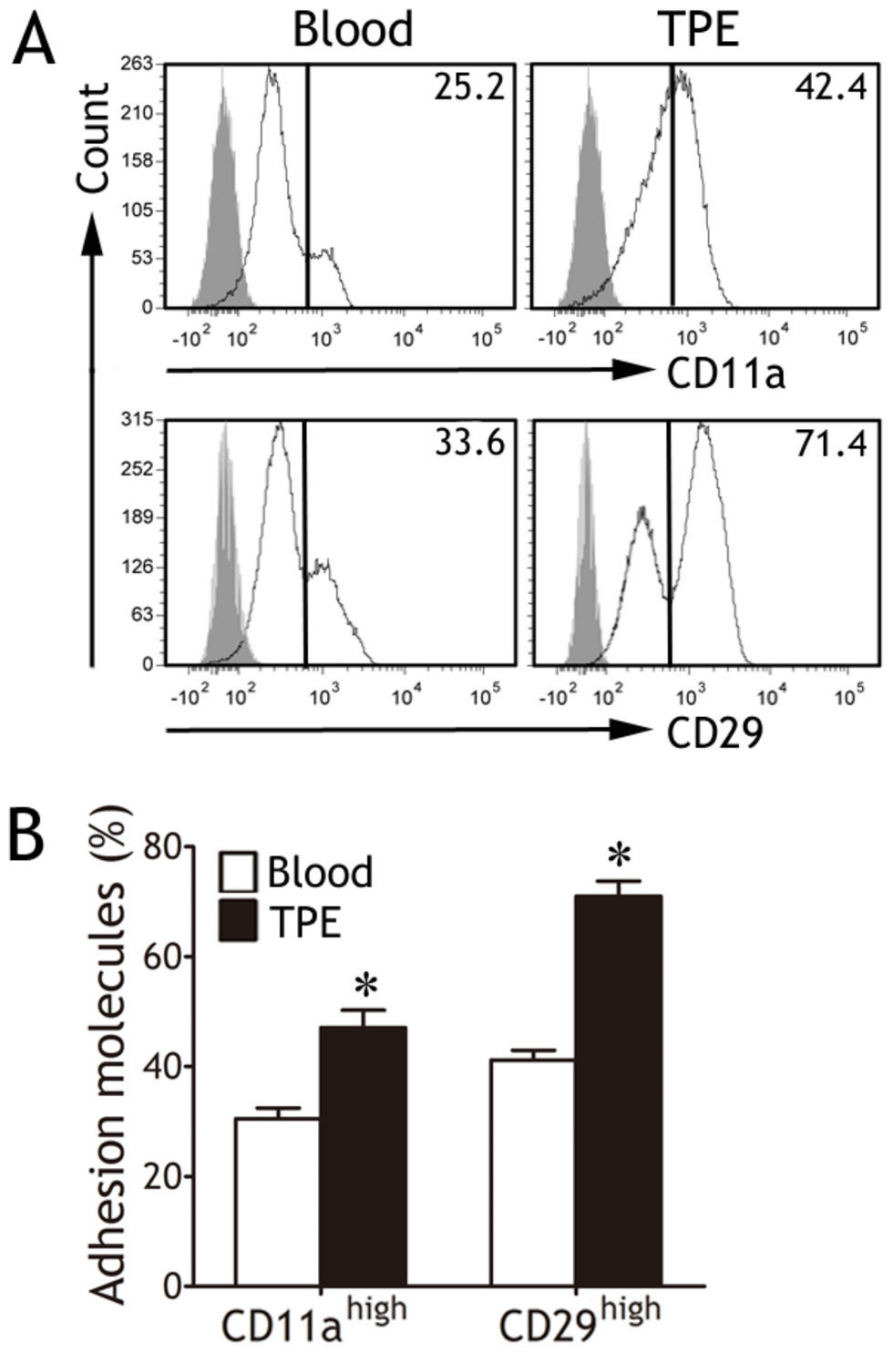
Expressions of CD11a and CD29 on CD4^+^ T cells. (A) Representative flow cytometric histograms show CD11a (upper panels) and CD29 (lower panels) expressions on CD4^+^ T cells from blood and tuberculous pleural effusion (TPE). According to expression intensities of the two adhesion molecules, CD4^+^ T cells were divided into two groups, respectively. (B) Comparisons of percentages of CD11a^high^CD4^+^ T cells and CD29^high^CD4^+^ T cells are shown (n = 12). The results are reported as mean ± SEM. The comparisons were determined by a Wilcoxon signed-rank test. *p < 0.05 compared with blood.

### Expressions of CD11a and CD29 on CD4^+^ T cells

Since LFA-1 (CD11a/CD18) and VLA-4 (CD49d/CD29) act as major counter-receptors for ICAM-1 and VCAM-1, respectively, we investigated expressions of CD11a and CD29 on CD4^+^ T cells. As shown in Figure 3A, nearly all of CD4^+^ T cells from TPE and the corresponding blood expressed CD11a and CD29. We therefore divided CD4^+^ T cells into two proportions according to expression intensities, and compared the proportions of CD11a^high^CD4^+^ T cells or CD29^high^CD4^+^ T cells in TPE and peripheral blood, respectively. We noted that CD11a^high^CD4^+^ T cells in TPE (47.1 ± 3.2%, n = 12) significantly increased as compared with peripheral blood (30.5 ± 1.9%, n = 12; p < 0.001, Wilcoxon signed-rank test) (Figure 3). Similarly, the proportion of CD29^high^CD4^+^ T cells in TPE (71.0 ± 2.7%) was also higher than that in blood (41.2 ± 1.8%, p < 0.001) (Figure 3).

**Figure 4:**
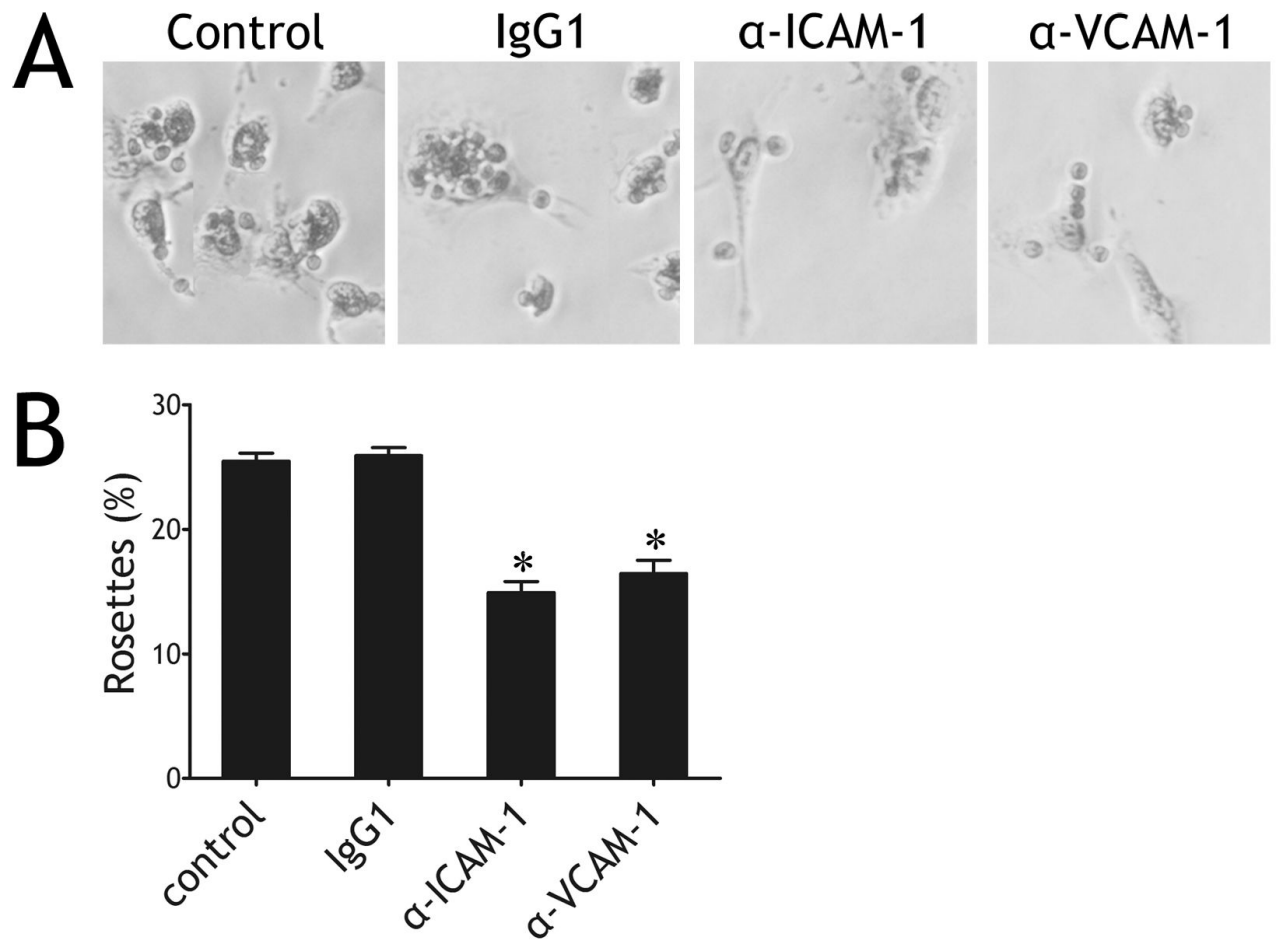
Intercellular adhesion molecule-1 (ICAM-1) and vascular cell adhesion molecule-1 (VCAM-1) expressed on pleural mesothelial cells (PMCs) were involved in the adhesion of CD4^+^ T cells. Pleural mesothelial cells isolated from tuberculous pleural effusion were cocultured with autologous CD4^+^ T. In some experiments, pleural mesothelial cells were preincubated with anti‒ICAM-1, ‒VCAM-1 mAb, or an irrelevant isotype control (IgG1). 48 h later, nonadherent cells were gently removed by washing and the slides were stained with Wright staining. (A) Representative photomicrographs of CD4^+^ T cells adhering to pleural mesothelial cells. Original magnification: × 400. (B) Summary percentages of CD4^+^ T cell-pleural mesothelial cell rosettes. The results are reported as mean ± SEM from 5 independent experiments. The comparisons were determined by Kruskal-Wallis one-way analysis of variance on ranks. *p < 0.05 compared with control group or isotype IgG1.

### ICAM-1/VCAM-1 Pathways Mediated CD4^+^ T Cell-PMC Adhesion

As shown in Figure 4, when PMCs derived from TPE were not preincubated with mAbs or irrelevant isotype IgG1, 25.5 ± 0.6% or 25.9 ± 0.7% of PMCs formed rosettes with CD4^+^ T cells, respectively; when PMCs were preincubated with anti‒ICAM-1 or ‒VCAM-1 mAb, 14.9 ± 0.9% or 16.4 ± 1.1% formed rosettes, respectively (both p < 0.05 compared with IgG1 control). These data showed that either anti‒ICAM-1 or ‒VCAM-1 mAb inhibited CD4^+^ T-PMC adhesion.

### ICAM-1/VCAM-1 Pathways Regulated CD4^+^ T Cell Activation, but not Proliferation

**Figure 5:**
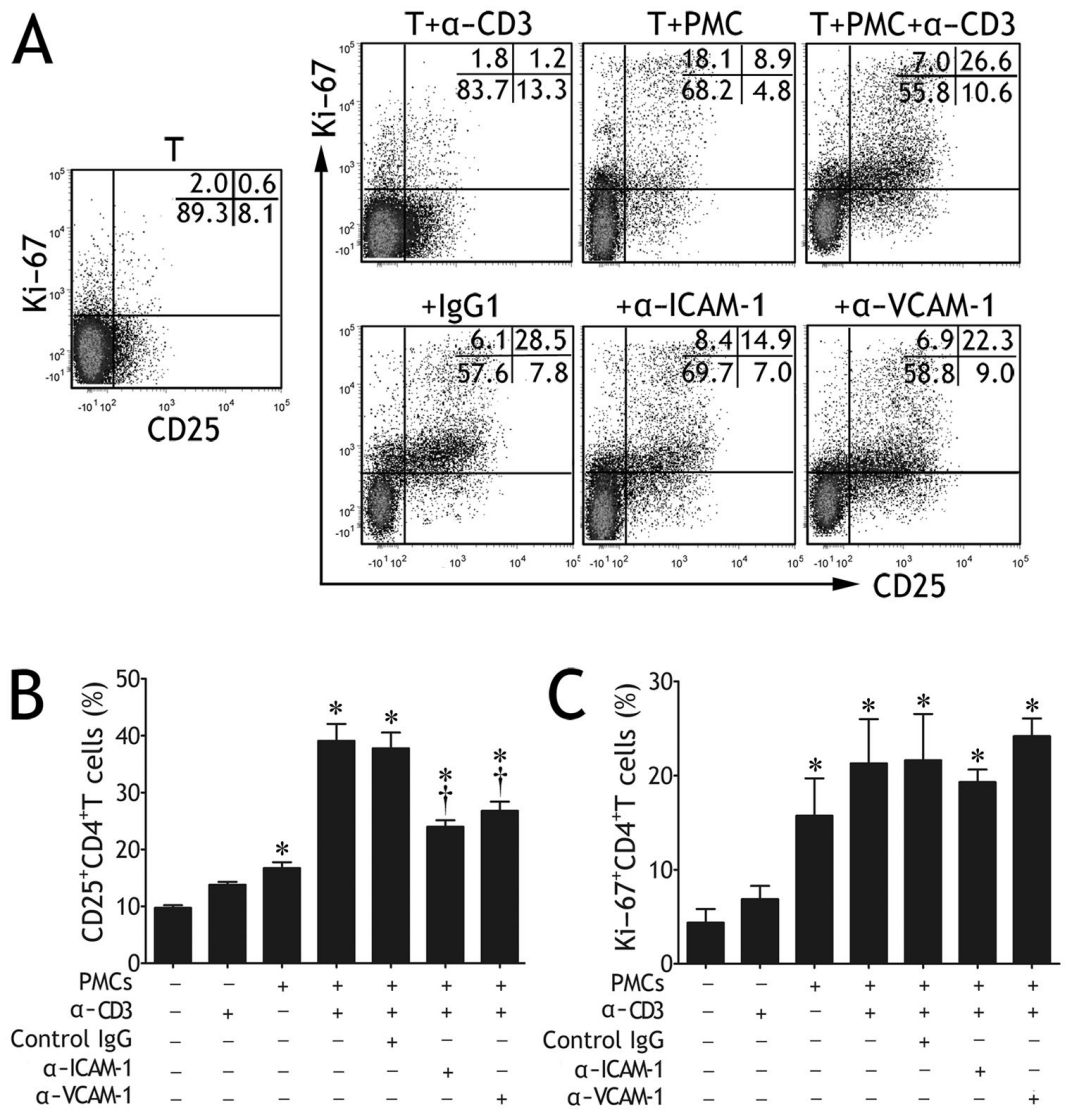
Intercellular adhesion molecule-1 (ICAM-1) and vascular cell adhesion molecule-1 (VCAM-1) expressed on pleural mesothelial cells (PMCs) were involved in CD4^+^ T cell activation, but not proliferation. Purified CD4^+^ T cells from tuberculous pleural effusion were cultured for 5 d in medium alone (T), or in the presence of anti‒CD3 mAb (T + α-CD3), or in the presence of autologous PMCs without or with anti‒CD3 mAb (T + PMC and T + PMC + α-CD3, respectively). In some cocultures of CD4^+^ T cells and PMCs plus anti‒CD3 mAb, PMCs were pre-incubated with anti‒ICAM-1 (α‒ICAM-1), ‒VCAM-1 (α‒VCAM-1) mAb, or an irrelevant isotype control (IgG1). (A) Representative flow cytometric dot plots show the percentages of Ki-67 ^+^ CD4^+^ T or CD25^+^CD4^+^ T cells. (B) Comparisons of percentages of CD25^+^CD4^+^ T cells are shown. (C) Comparisons of percentages of Ki-67 ^+^ CD4^+^ T cells are shown. In panels B and C, the results are reported as mean ± SEM from 4 independent experiments. The comparisons were determined by Kruskal-Wallis one-way analysis of variance on ranks. *p < 0.05 compared with medium control, † p < 0.05 compared with irrelevant IgG control.

Our previous studies have demonstrated that TPE derived-PMCs functioned as antigen presenting cells to stimulate CD4^+^ T cell proliferation and Th cell differentiation [8,9]. Considering that ICAM-1 or VCAM-1 mediated adhesion between CD4^+^ T cells and PMCs, we wondered whether such an adhesion facilitated the formation of tight junction between CD4^+^ T cells and PMCs, thus promoted the activation and proliferation of T cells stimulated by PMCs. To address this, we cocultured PMCs with autologous CD4^+^ T cells isolated from TPE, using CD25 and Ki-67 expressions to indicate the activation and proliferation of CD4^+^ T cells, respectively. Consistent with our previous data [8,9], PMCs significantly promoted CD4^+^ T cell activation and proliferation, especially in the presence of anti‒CD3 mAb (Figure 5). The novel findings of our current study were that prestimulation of PMCs with anti‒ICAM-1 or ‒VCAM-1 mAb significantly inhibited CD4^+^ T cell activation induced by PMCs, but did not affect CD4^+^ T cell proliferation (Figure 5).

### ICAM-1/VCAM-1 Pathways Regulated Selective CD4^+^ T Cell Expansion

**Figure 6:**
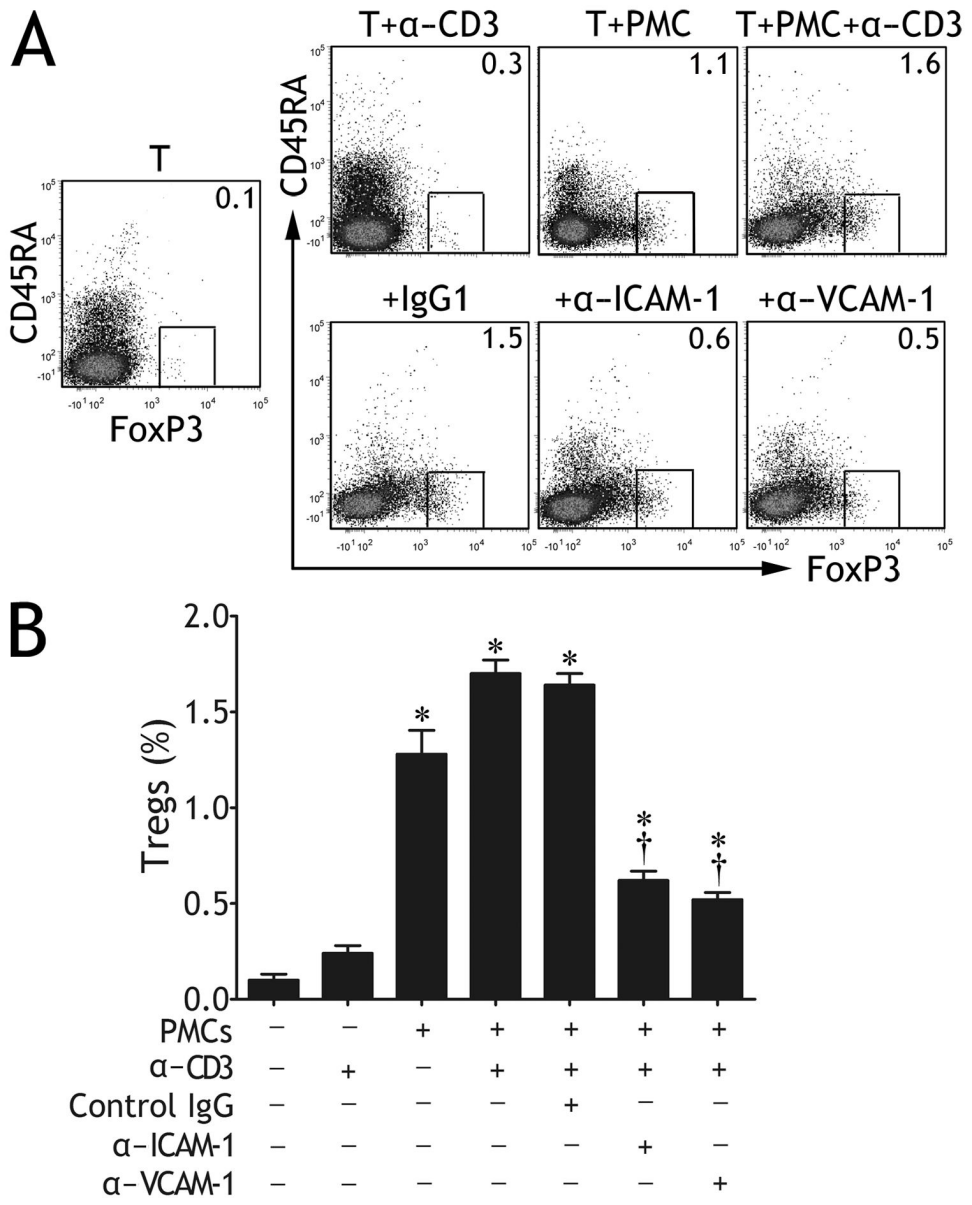
Intercellular adhesion molecule-1 (ICAM-1) and vascular cell adhesion molecule-1 (VCAM-1) expressed on pleural mesothelial cells (PMCs) were involved in expansion of effector regulatory T cells (eTregs). Purified CD4^+^ T cells from tuberculous pleural effusion were cultured for 5 d in medium alone (T), or in the presence of anti‒CD3 mAb (T + α-CD3), or in the presence of autologous PMCs without or with anti‒CD3 mAb (T + PMC and T + PMC + α-CD3, respectively). In some cocultures of CD4^+^ T cells and PMCs plus anti‒CD3 mAb, PMCs were pre-incubated with anti‒ICAM-1 (α‒ICAM-1), ‒VCAM-1 (α‒VCAM-1) mAb, or an irrelevant isotype control (IgG1). eTregs were identified by intracellular FoxP3 staining. (A) Representative flow cytometric dot plots show FoxP3 expression in CD45RA^-^ CD4^+^ T cells. (B) Comparisons of percentages of FoxP3 ^high^CD45RA^-^CD4^+^ T cells in each group. The results are reported as mean ± SEM from 5 independent experiments. The comparisons were determined by Kruskal-Wallis one-way analysis of variance on ranks. *p < 0.05 compared with medium control, † p < 0.05 compared with irrelevant isotype IgG control.

We next cocultured PMCs with autologous CD4^+^ T cells purified from TPE in the presence of anti‒CD3 mAb; in some experiments, PMCs were preincubated with anti‒ICAM-1, ‒VCAM-1 mAb, or irrelevant isotype IgG1. Consistent with our previous report [8,9], we confirmed once again that PMCs significantly promoted the expansions of Th1, Th9, Th17 and Th22 cells (data not shown). The novel findings in the present study were that PMCs promoted the expansion of CD45RA^-^FoxP3 ^high^CD4^+^ T cells from CD4^+^ T cells (Figure 6). CD45RA^-^FoxP3 ^high^CD4^+^ T cells were reported to be effector Tregs (eTregs), which possessed potent suppressive activity [14]. Furthermore, anti‒ICAM-1 or ‒VCAM-1 mAb significantly inhibited eTreg expansion induced by PMCs (Figure 6), however, these mAbs did not affect Th1, Th9, Th17, or Th22 cell expansions (data not shown).

## Discussion

PMCs are an important component of the pleural environment, they may collaborate with the other kinds of cells, including CD4^+^ T cells, in the generation of local cell-mediated immunity to various pathogens, including MTB. Actually, migration of CD4^+^ T cells from peripheral blood to pleural space is a key feature of TPE [3]. Adhesion molecules, such as ICAM-1 and VCAM-1, expressed on the surface of various cell types are of importance in cell-to-cell interactions. It has been reported that ICAM-1 expression was upregulated on PMCs in patients with TPE [11] and ICAM-1 played a critical role in leukocyte trafficking from vascular compartment into TPE [11,15]. We noted in the present study that PMCs from TPE, expressed high levels of ICAM-1 and moderate levels of VCAM-1, and that the percentages of ICAM-1-positive or VCAM-1-positive PMCs in TPE were significantly higher than those of PMC line. Our data confirmed that MTB infection upregulated the expression extents of adhesion molecules on PMCs.

Early studies have demonstrated that cultured human mesothelial cells expressed appreciable levels of ICAM-1 and VCAM-1, and they were increased by *in vitro* exposure to some cytokines, including IFN-γ [16]. It has been well documented that TPE was enriched with CD4^+^CDw29^+^ T cells, which are thought to represent “memory” T cells, and these pleural CD4^+^CDw29^+^ cells, but not CD4^+^CDw29^-^ cells, proliferated vigorously and produced high levels of IFN-γ when stimulated with purified protein derivative of MTB [17]. High concentration of IFN-γ could always be found in TPE and served as a reasonable diagnostic biomarker for TPE [18]. Our current data showed that exogenous IFN-γ notably enhanced ICAM-1 MFI of PMCs *in vitro*, while TPE didn’t increased ICAM-1 MFI. On the other hand, the expression of VCAM-1 on TPE derived-PMCs was significantly upregulated by IL-4 and downregulated by TGF-β.

We further determined the expressions of major counter-receptors, CD11a and CD29, for ICAM-1 and VCAM-1, respectively, on CD4^+^ T cells. It has been reported that integrins existed on cell surface mainly in an inactive form until they received stimulating signals, such as those induced by chemokine receptors or T cell receptors [19], and that CD11a^high^ or CD29^high^CD4^+^ T cells stood for memory or previously activated cells [20]. Animal studies suggested that CD11a was required for protective immunity during pulmonary tuberculosis infection [21]. Feng et al [22] reported that compared with uninfected mice, the percentage of lung CD29^high^CD4^+^ T cells increased in mice infected with MTB, and these cells were activated/memory T cells and capable of producing IFN-γ. Consistent with previous studies, we found that CD4^+^ T cells both in TPE or blood mostly expressed CD11a or CD29, and the percentages of CD11a^high^CD4^+^ T cells or CD29^high^CD4^+^ T cells in TPE were higher than those in blood. More importantly, we have further demonstrated that PMCs mediated CD4^+^ T cell adhesion via ICAM-1‒or VCAM-1‒dependent mechanisms.

ICAM-1/LFA-1 pathway has been known to enhance T cell-antigen presenting cell interactions and to promote T cell proliferation and activation [23]. Earlier studies reported that ICAM-1 acted as dominant costimulator in the absence of B7/CD28 costimulation, and the blockade of ICAM-1 elicited complete inhibition of CD4^+^ T cell proliferation and activation [24]. Our data showed that blocking ICAM-1 or VCAM-1 pathways on PMCs partly prevented CD4^+^ T cell activation, but not proliferation. The high expression levels of CD80/CD86 costimulatory molecules on PMCs [8,9] might partly result in the discrepancies.

Our previous data have demonstrated that PMCs derived from TPE promoted proliferation of naïve CD4^+^ T cell and differentiation of Th9, Th22, Th17 and Th1 cells with or without the stimulation of MTB-specific antigens [8,9]. Our present study replaced MTB-specific antigens with anti-CD3 mAb and extended the previous findings, showing that ICAM-1- or VCAM-1-dependent pathways facilitated eTreg expansion stimulated by PMCs in response to non-specific TCR stimulation, since blockade of the anti‒ICAM-1 or ‒VCAM-1 mAb inhibited such an expansion. There were still controversies in Th subset selective expansion mediated by adhesion molecules so far. Salomon et al [25] reported that ICAM-1/LFA-1 interaction inhibited Th2 cytokine production; whereas Takamoto et al [26] showed that ICAM-1/LFA-1 interaction was important for IL-4 production, and VCAM-1/VLA-4 interaction was important for IL-5 production. Further studies suggested that ICAM-1/LFA-1 ligation favors human Th1 development [27,28]. Recently, it has been demonstrated that ICAM-1/LFA-1 stimulated human and mouse T cells refractory to TGF-β-mediated induction of FoxP3 and Th17 differentiation [29], while some other studies supported our results showing that Treg amplification was dependent on ICAM-1 [30,31], and deficiency of ICAM-1 might result in overwhelming inflammation [30]. We considered that the origin or activatory state of antigen presenting cells, the responder T cell density, and the doses of anti‒ICAM-1 or ‒VCAM-1 mAb might account for the discrepancies, and further investigations were needed.

In conclusion, our data showed that PMCs in TPE expressed increased levels of ICAM-1 and VCAM-1. IFN-γ primarily promoted ICAM-1 expression and IL-4 promoted VCAM-1 expression. ICAM-1 and VCAM-1 acted as a double-edged sword, mediating adhesion and activation of CD4^+^ T cells, and selectively promoting eTreg expansion from CD4^+^ T cells stimulated by PMCs.

## Supporting Information

Figure S1 **Epidermal growth factor (EGF) didn’t affect expressions of ICAM-1 and VCAM-1 on pleural mesothelial cells (PMCs).** PMCs from tuberculous pleural effusion were cultured in medium alone (n = 12) or in the presence of EGF (20 ng/ml) (n = 3), and ICAM-1 and VCAM-1 expressions were detected. (A) Representative flow cytometric dot plots show ICAM-1 and VCAM-1 expressions on PMCs cultured in medium alone (upper plane), or in the presence of EGF (lower plane) (B.C). Comparisons of mean fluorescence intensity (MFI) of ICAM-1 and VCAM-1 on PMCs. The results are reported as mean ± SEM.
(TIF)

Figure S2 **Pleural mesothelial cells (PMCs) from transudative pleural effusion expressed lower levels of ICAM-1 and VCAM-1 than those from tuberculous pleural effusion.** A non-malignant transformed mesothelial cell line Met5A cell (PMC line, n = 12) or PMCs derived from tuberculous pleural effusion (TPE, n = 12), or PMCs derived from transudative pleural effusion (TE, n = 4) were stained using anti‒ICAM-1, ‒VCAM-1 mAb, or isotype control IgG. (A) Representative flow cytometric histogram plots show ICAM-1 and VCAM-1 expressions on PMCs. Light gray histograms indicate isotype controls (B.C). Comparisons of mean fluorescence intensity (MFI) of ICAM-1 and VCAM-1 on PMCs. The results are reported as mean ± SEM.
(TIF)
