# Supplementary figures and images for: Regulation of CD4+ T Cells by Pleural Mesothelial Cells via Adhesion Molecule-Dependent Mechanisms in Tuberculous Pleurisy
Source: PLoS One. 2013 Sep 19;8(9):e74624. doi: 10.1371/journal.pone.0074624 (PMC3777994; doi:10.1371/journal.pone.0074624)

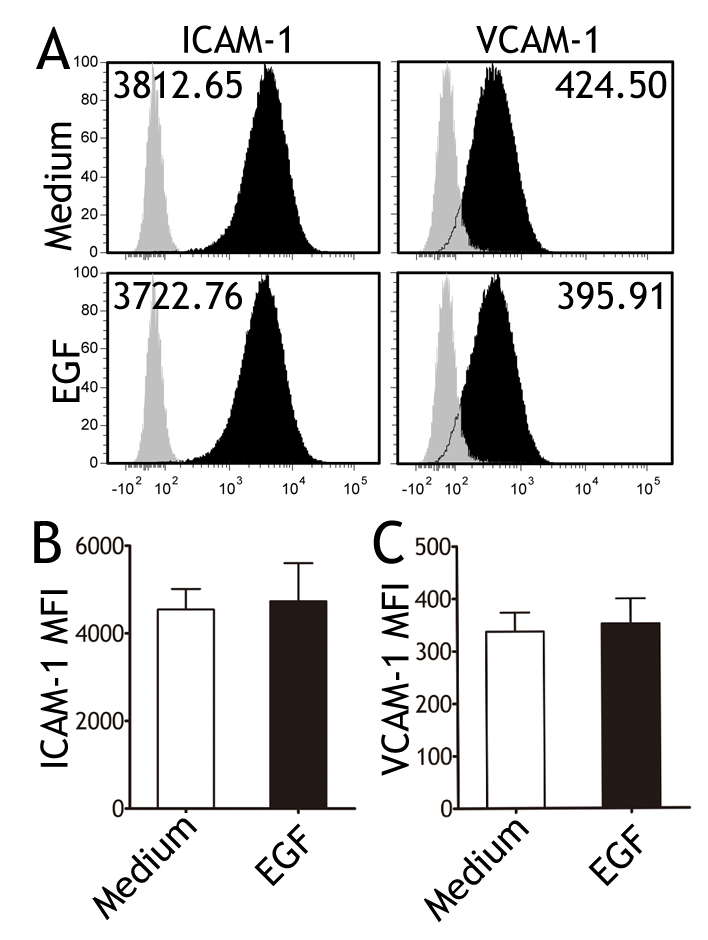

Supplement: Figure S1 — Epidermal growth factor (EGF) didn’t affect expressions of ICAM-1 and VCAM-1 on pleural mesothelial cells (PMCs). PMCs from tuberculous pleural effusion were cultured in medium alone (n = 12) or in the presence of EGF (20 ng/ml) (n = 3), and ICAM-1 and VCAM-1 expressions were detected. (A) Representative flow cytometric dot plots show ICAM-1 and VCAM-1 expressions on PMCs cultured in medium alone (upper plane), or in the presence of EGF (lower plane) (B.C). Comparisons of mean fluorescence intensity (MFI) of ICAM-1 and VCAM-1 on PMCs. The results are reported as mean ± SEM. (TIF) [file pone.0074624.s001.tif]

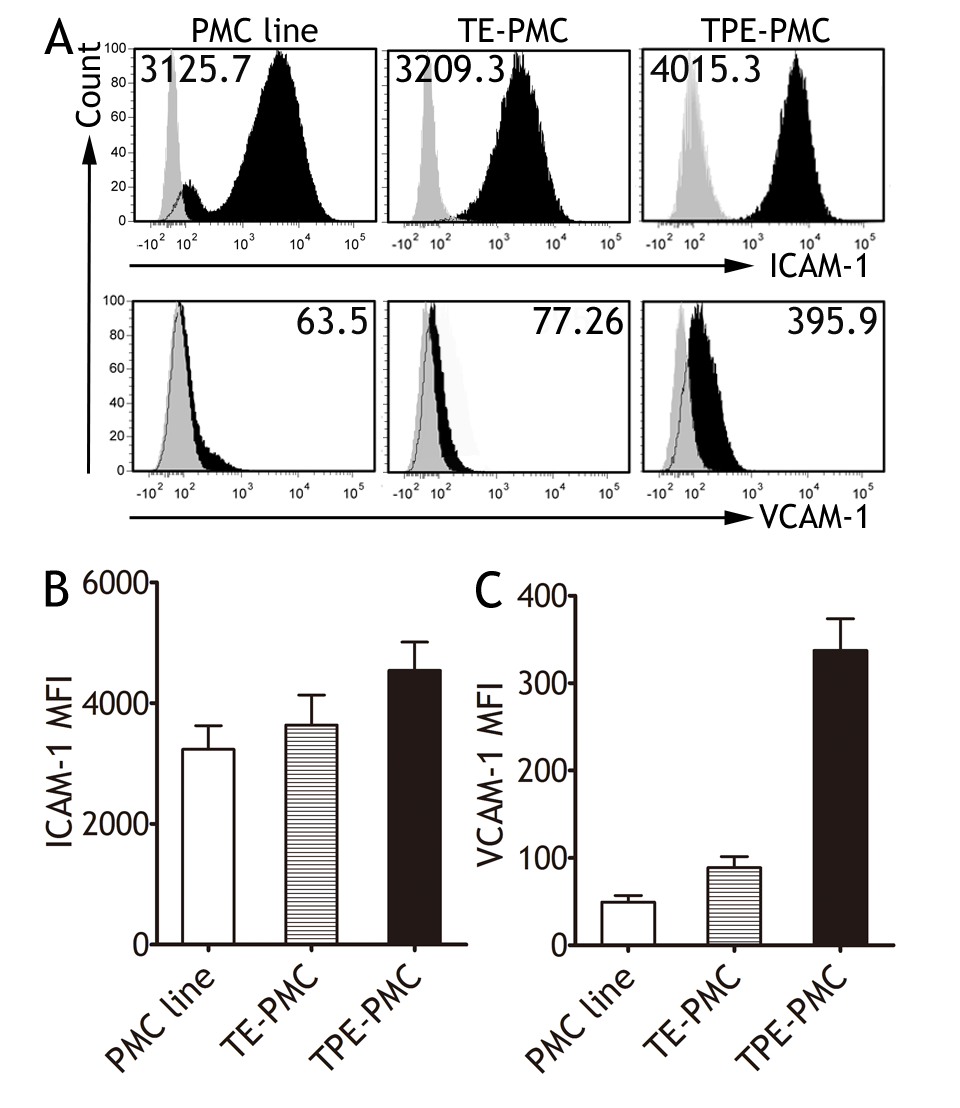

Supplement: Figure S2 — Pleural mesothelial cells (PMCs) from transudative pleural effusion expressed lower levels of ICAM-1 and VCAM-1 than those from tuberculous pleural effusion. A non-malignant transformed mesothelial cell line Met5A cell (PMC line, n = 12) or PMCs derived from tuberculous pleural effusion (TPE, n = 12), or PMCs derived from transudative pleural effusion (TE, n = 4) were stained using anti‒ICAM-1, ‒VCAM-1 mAb, or isotype control IgG. (A) Representative flow cytometric histogram plots show ICAM-1 and VCAM-1 expressions on PMCs. Light gray histograms indicate isotype controls (B.C). Comparisons of mean fluorescence intensity (MFI) of ICAM-1 and VCAM-1 on PMCs. The results are reported as mean ± SEM. (TIF) [file pone.0074624.s002.tif]
